# The Efficacy of Calcium Sulfate/Hydroxyapatite (CaS/HA) Gentamicin in Osteomyelitis Treatment: A Case Series

**DOI:** 10.3390/antibiotics13111068

**Published:** 2024-11-10

**Authors:** Amir Human Hoveidaei, Sanoj Shahul, Sina Esmaeili, Kasra Pirahesh, Amirhossein Ghaseminejad-Raeini, Abijith Annasamudram, Raj Krishna Shrestha, Janet D. Conway

**Affiliations:** 1 International Center for Limb Lengthening, Rubin Institute for Advanced Orthopedics, Sinai Hospital of Baltimore, Baltimore, MD 2125, USA; 2 Sina University Hospital, Tehran University of Medical Sciences, Tehran 1416753955, Iran

**Keywords:** osteomyelitis, antibiotic, calcium sulfate/hydroxyapatite, CERAMENT G, infection eradication

## Abstract

Background: Osteomyelitis is a challenging condition caused by infection and inflammation of the bone, presenting a significant economic burden to healthcare systems. Calcium sulfate/hydroxyapatite (CaS/HA) is a bone void filler composed of 60% calcium sulfate and 40% hydroxyapatite. This case series aimed to report the efficacy and infection-related outcomes of CaS/HA combined with Gentamicin (CaS/HA-G) in treating osteomyelitis. Methods: Patients aged 18 and older diagnosed with osteomyelitis requiring surgical intervention and treated with CaS/HA-G during their procedure were included in the study, with a median (Q1–Q3) = 10 (7–16)-month follow-up period of time. Data collected included demographic, surgical, and outcome information. Infection eradication was determined by the normalization of the C-reactive protein, erythrocyte sedimentation rate levels, or the absence of clinical infection symptoms. Results: The case series involved 21 patients (twelve male, nine female) with a mean (SD) age of 54.8 (16.6) years. Vancomycin or/and Tobramycin were used as an additional antibiotic in 17 patients. At the last follow-up, 20 out of 21 patients (95.2%) had eradicated the infection, with a median (Q1–Q3) eradication time of 128 (71.8–233.5) days. Conclusions: In conclusion, this study demonstrates that CaS/HA-G is effective in controlling osseous infection in osteomyelitis while acting as an absorbable bone void filler.

## 1. Introduction

Osteomyelitis is a challenging condition arising from infection and inflammation of the bone. The overall annual incidence of osteomyelitis is estimated to be 21.8 cases per 100,000 in the US, with an increasing trend during the past four decades [1]. Studies in other nations have similarly reported an increasing fashion in the prevalence of osteomyelitis [2,3]. In addition, the aging population and the projected increase in the global prevalence of diabetes pose a significant increase to the potential burden of osteomyelitis [1]. With current management strategies, the economic burden of osteomyelitis is substantial in healthcare [4,5].

Despite advances in surgical and medical treatment, success rates still vary between 70 to 90% in fracture-related infections, with recurrence in 6–9% of patients [6,7]. A wide set of medical and surgical treatment options are available. Yet, guidelines suggest that combined treatment approaches should be considered in all cases, especially in patients with chronic osteomyelitis [8]. Proper dead space management and subsequent reconstruction are critically important in the surgical treatment of osteomyelitis. Although many dead space management strategies currently exist, no single strategy stands superior to any other, and the ideal void filler is yet to be found [9].

Calcium sulfate/hydroxyapatite (CaS/HA) is a bone void filler composed of 40% hydroxyapatite and 60% calcium sulfate [10]. Recent studies have highlighted the capability of CaS/HA combined with Gentamicin (CaS/HA-G), commercially available as CERAMENT G, in treating infections with Gentamicin-resistant bacteria, arising from the high local concentrations of the antibiotic agent [11]. With preliminary investigations of CaS/HA-G showing comparable results to autologous bone grafts [12,13], in this case series, we aimed to report the efficacy and infection-related outcomes of CaS/HA-G in treating patients with osteomyelitis.

## 2. Results

The case series included 22 patients who underwent orthopedic procedures using CaS/HA-G. However, one patient was excluded due to a loss of follow-up following their death from cancer, resulting in a total of 21 patients analyzed. Among them, there were twelve male and nine female patients, with a mean (SD) age of 54.8 (16.6) years. Four patients had a history of diabetes mellitus, and two were smokers. The median (Q1–Q3) follow-up duration was 10 (7–16) months. The median (Q1–Q3) volume of CaS/HA-G used was 20.0 (12.5–40.0) cc. The most commonly infected sites were the tibia (nine cases) and the femur (five cases). Vancomycin or/and Tobramycin were used as an additional antibiotic in 17 patients. The remaining patients did not receive any additional antibiotics. Further baseline details, including preoperative diagnoses, are presented in Table 1.

Three cases (Nos. 1, 11, and 13) experienced reinfection and needed reoperation, while one additional case (No. 7) also required reoperation. Case 1 involved a 40-year-old female who underwent a partial resection of the tibia for osteomyelitis. A 60 cc CaS/HA-G was inserted to fill the defect, and a medial reverse hemisoleus flap with a split-thickness skin graft (STSG) was applied to the muscle flap, measuring 9 × 4 cm. She presented with a draining medial wound and acute sepsis. After 5 weeks from the first surgery, diagnosed with right distal tibia/ankle osteomyelitis with acute sepsis, a retained posterior plate, and wound dehiscence, she underwent a reoperation at the sixth week. The procedures included the removal of the posterior ankle arthrodesis plate, another partial resection of the tibia for osteomyelitis, insertion of an antibiotic-coated ankle fusion rod for prophylaxis due to medial and posterior tibial defects, and complex wound closure with readvancement of the medial hemisoleus muscle flap, and application of a wound vac. Case 11 involved a 57-year-old male who underwent resection of osteomyelitis and insertion of CaS/HA-G with vancomycin. However, approximately four months later, he needed right tibial wound debridement and a skin graft. Case 13 concerned a 67-year-old diabetic male with a periprosthetic infection following a total knee arthroplasty (TKA). The TKA device was removed, and he underwent knee arthrodesis with a long rod. Four months later, he required repair of a nonunion knee fusion using a plate and an antibiotic-coated rod (Table 2).

**Figure 1:**
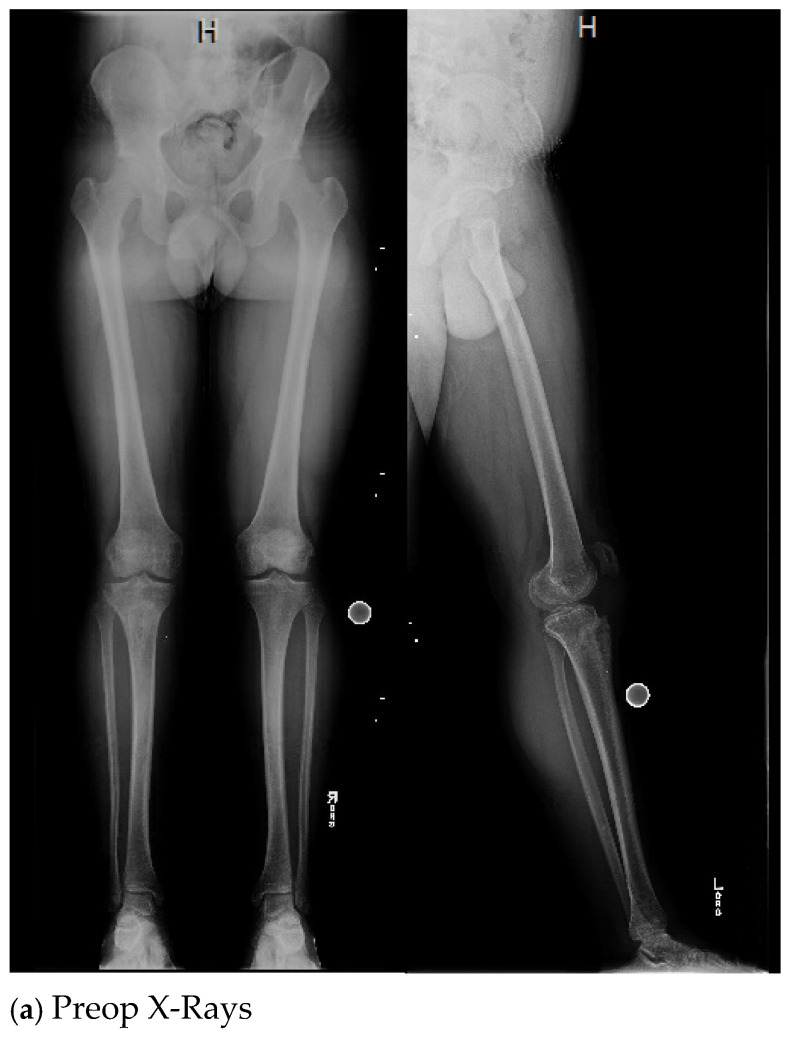

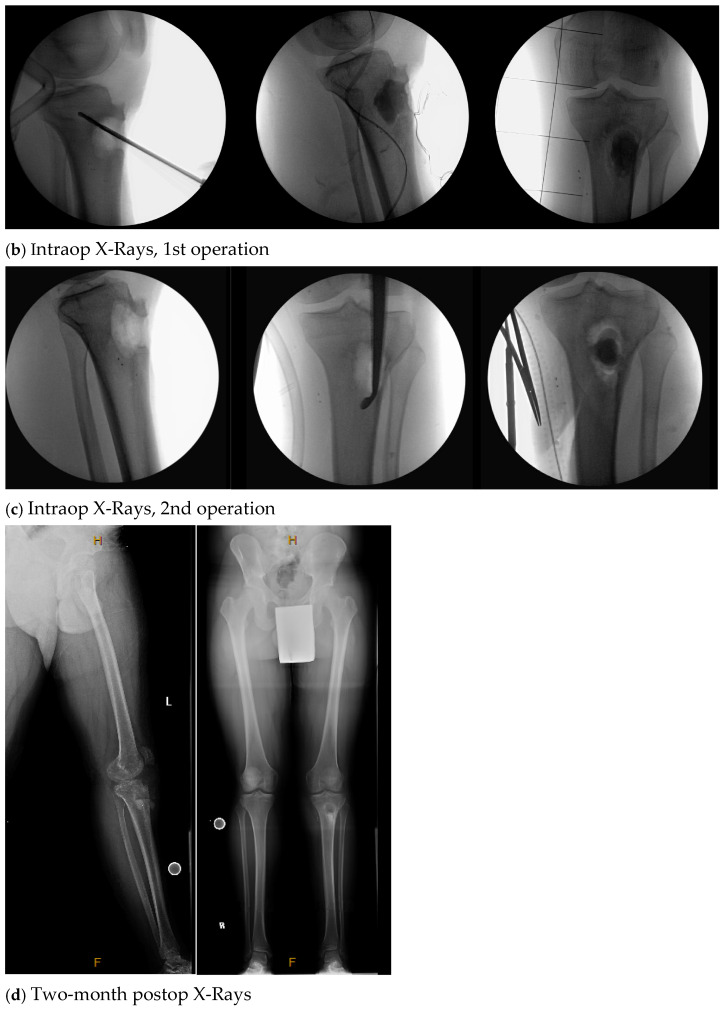

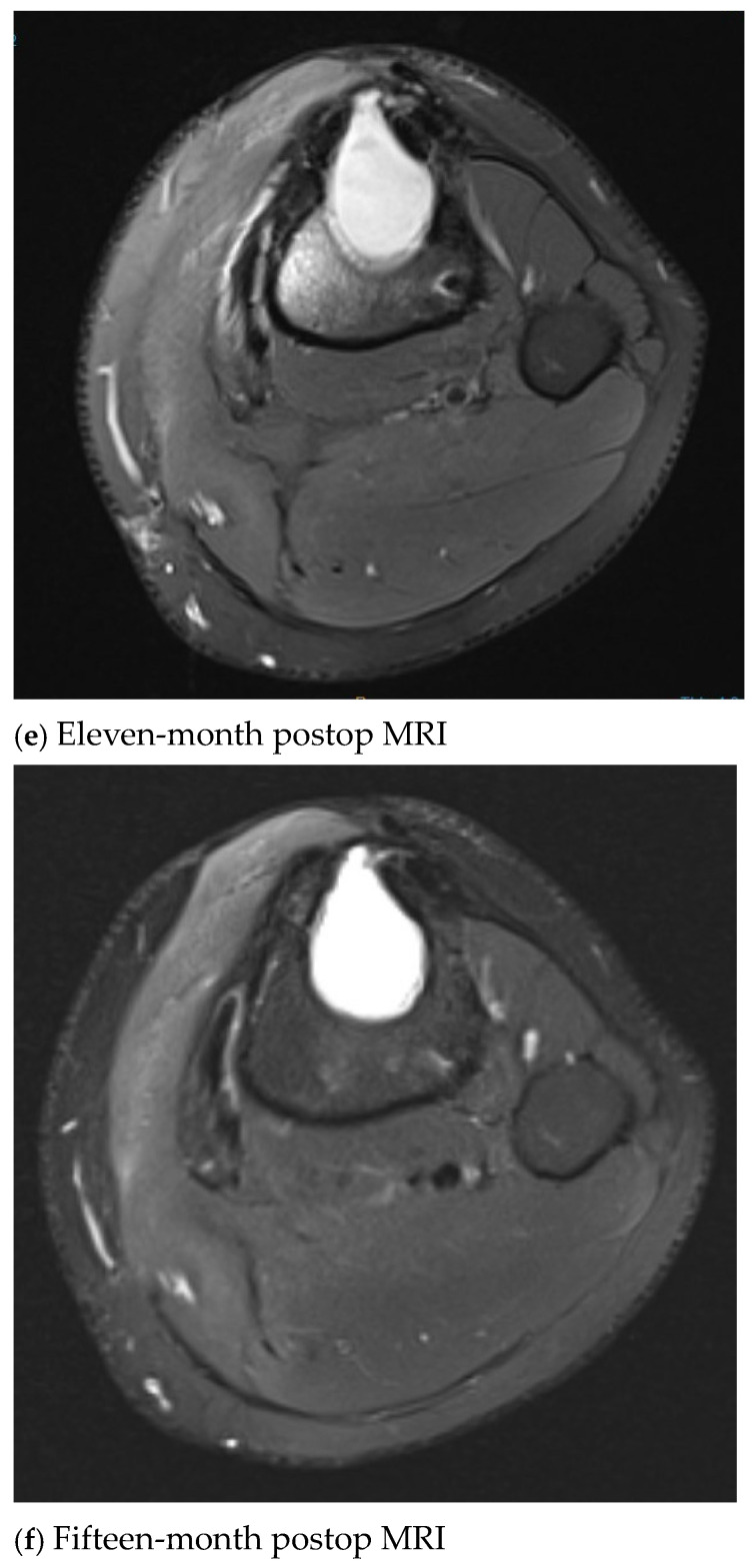
Imaging studies of case 7.

Case 7 was reoperated due to continuing drainage from a small area of dehiscence/nonhealing, and it was decided the best next course would be surgical washout/debridement with application of a medial gastrocnemius muscle flap and skin graft application with a 20 cc insertion of CaS/HA-G. Figure 1 shows the imaging studies on case 7.

In 18 out of 21 cases with available laboratory data, the median (Q1–Q3) Erythrocyte sedimentation rate (ESR) was 21 (10.5–28.3) mm/h based on their latest tests. Based on the last follow-up, twenty out of twenty one patients (95.2%) had eradicated the infection, with a median eradication time of nearly 4 months—median (Q1–Q3) eradication time = 128 (71.8–233.5) days. Table 2 presents the tissue culture findings, revealing that Staphylococcus was the most commonly isolated organism, identified in 10 cases. The infection persisted in case 5 at the 16-month follow-up. This case had a device removal through revision TKA with the insertion of a knee fusion nail due to a failed right TKA with infection. CaS/HA-G, vancomycin, and tobramycin were also used (Table 2).

## 3. Discussion

In the current case series, we demonstrated the efficacy of CaS/HA-G in treating patients with osteomyelitis across a variety of infected sites. Overall, CaS/HA-G performed well both in controlling the osteomyelitis and in serving as an effective bone void feeling structure. These outcomes are primarily attributable to its integrated calcium sulfate and hydroxyapatite structure, which effectively releases the antibiotic Gentamicin to the local tissue.

As an essential step in managing chronic osteomyelitis, extensive necrotic tissue debridement results in dead spaces prone to getting filled with hematoma or seroma collections [14]. Managing these dead spaces remains a critical area of research in the surgical treatment of osteomyelitis [9,15]. Previously, a two-stage method consisting of inserting a polymethyl methacrylate (PMMA) and then replacing it with a natural bone graft was applied [9]. However, the recent literature focuses on a single-stage approach using materials with three key properties: sufficient antibiotic delivery, adequate void filling, and bioabsorbability [16]. Theoretically, CaS/HA-G possesses all these properties, while our findings suggest that these properties are also evident and effective in clinical practice.

The majority of our cases yielded positive Staphylococcus cultures, consistent with the most commonly isolated organisms reported in the bone- and joint-infection literature [17]. Notably, we encountered four cases of Methicillin-resistant Staphylococcus aureus, which is known to be more challenging to manage compared to other infections [18,19,20,21]. Our treatment showed a promising result in the eradication of these Methicillin-resistant Staphylococcus aureus cases. However, it is noteworthy that in two out of the three reinfection cases, a positive culture for Serratia, a Gram-negative species, was identified, underscoring the need for further consideration of antibiotic options.

In our study, there were only three cases of reinfection and one case of noneradication. CaS/HA-G provides a biphasic local antibiotic delivery of Gentamicin, which can easily reach the minimum inhibitory concentration and minimum biofilm eradication concentration for many organisms [22,23]. The slow, consistent, and high local antibiotic delivery prevents the risk of systemic toxicity posed by parenteral antibiotics [23,24]. Interestingly, the dissolution time and type of the local antibiotic carrier seems to impact the outcomes. This is highlighted by Ferguson et al. showing a better radiological and clinical outcome for CaS/HA-G when comparing it to a similar void filler, Osteoset T [25].

We observed four cases of reoperation. Unlike similar synthetic void feeling materials, CaS/HA-G provides a structure that can be converted into bone tissue. As the substance dissolves, the hydroxyapatite acts as an osteoconductive carrier and promotes bone healing [26]. This remarkably decreases the need for subsequent surgeries to replace the void feeling substance with a bone graft, potentially reducing both the patient burden and the economic costs.

An important consideration in the reoperation cases is the site of infection. The anteromedial section of tibia, mainly due to inadequate soft tissue coverage, appeared more prone to surgical complications. This area often requires larger muscle flaps for covering the defects. Notably, adequate soft tissue coverage is crucial for proper perfusion to the regeneration site and effective delivery of other systemic antibiotics [16,27,28]. The size of the bone void and the higher amount of CaS/HA-G used in these cases could be other reasons contributing to the need for a reoperation (like cases 1 and 11 with ≥50 cc CaS/HA-G). Furthermore, the timing of the reoperation warrants attention, as we observed a relatively late alleviation of swelling in one case suspected of needing surgical drainage beforehand. We suspect that a less aggressive strategy in managing drainage cases may help prevent additional surgical interventions.

Overall, our findings align with those of previously published studies from other regions around the world [22,25,29]. However, this is the largest study to evaluate the efficacy of CaS/HA-G for treating osteomyelitis in the United States since the FDA approved CERAMENT G in May 2022. However, our study has certain limitations. Firstly, a median follow-up duration of 10 months may fall short in detecting all complications; thus, future studies should consider a longer follow-up period. No functional scores were presented and the focus of our study was on infection-related outcomes. Also, we had a relatively diverse set of cases with osteomyelitis across various sites, which may introduce variability in the outcomes.

## 4. Conclusions

In conclusion, we demonstrated that CaS/HA-G may be effective in controlling osseous infection in osteomyelitis while acting as an absorbable bone void filler. CaS/HA-G provided adequate structural support while reducing the need for subsequent surgeries. Considering that we used an injectable paste form of CaS/HA-G, we recommend further studies to investigate and compare the efficacy of its bead form as well. Further research should also explore the optimal amount of CaS/HA-G and strategies for managing drainage.

## 5. Materials and Methods

### 5.1. Patient Involvement

This retrospective case series study evaluates the outcomes of osteomyelitis patients who underwent a surgical procedure incorporating the use of CaS/HA-G (CERAMENT G). Patients aged 18 and older diagnosed with osteomyelitis requiring surgical intervention and who received CaS/HA-G during their surgical procedure were included in the study. Some of the included patients had infections at the sites of previous orthopedic surgeries. Patients were excluded if they were under 18 years of age, had incomplete medical records, or were lost to follow-up before the minimum period of 6 months. The procedures were performed by the senior author at Sinai Hospital in Baltimore between October 2022 and February 2024. All selected patients were followed up with until September 2024, with a minimum follow-up period of six months. Ethical approval for this study was obtained from the LifeBridge Health Institutional Review Board (IRB), which determined that this project is exempt from Department of Health and Human Services regulations for the protection of human subjects.

### 5.2. Data Collection

To gather the demographic, surgical, and outcome information, patient records were reviewed. The data collected included patient age, sex, diabetes mellitus status, and smoking status. Surgical details covered the preoperative diagnosis, the type of surgery performed, the quantity of CaS/HA-G injected, and any additional antibiotics administered. The outcome measures included reinfection rates, reoperation rates, C-reactive protein (CRP) levels, ESR, and tissue culture findings. Patient records were accessed and reviewed by a designated team member to ensure accuracy.

### 5.3. Surgical Technique

**Figure 2:**
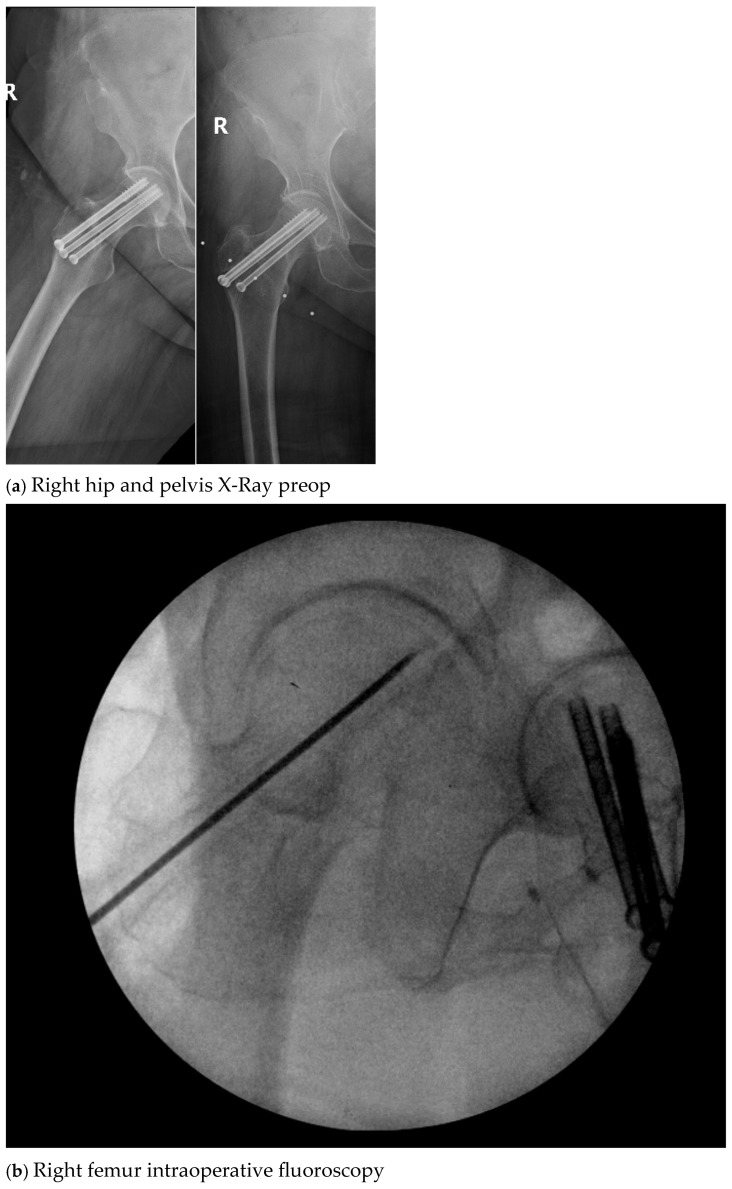

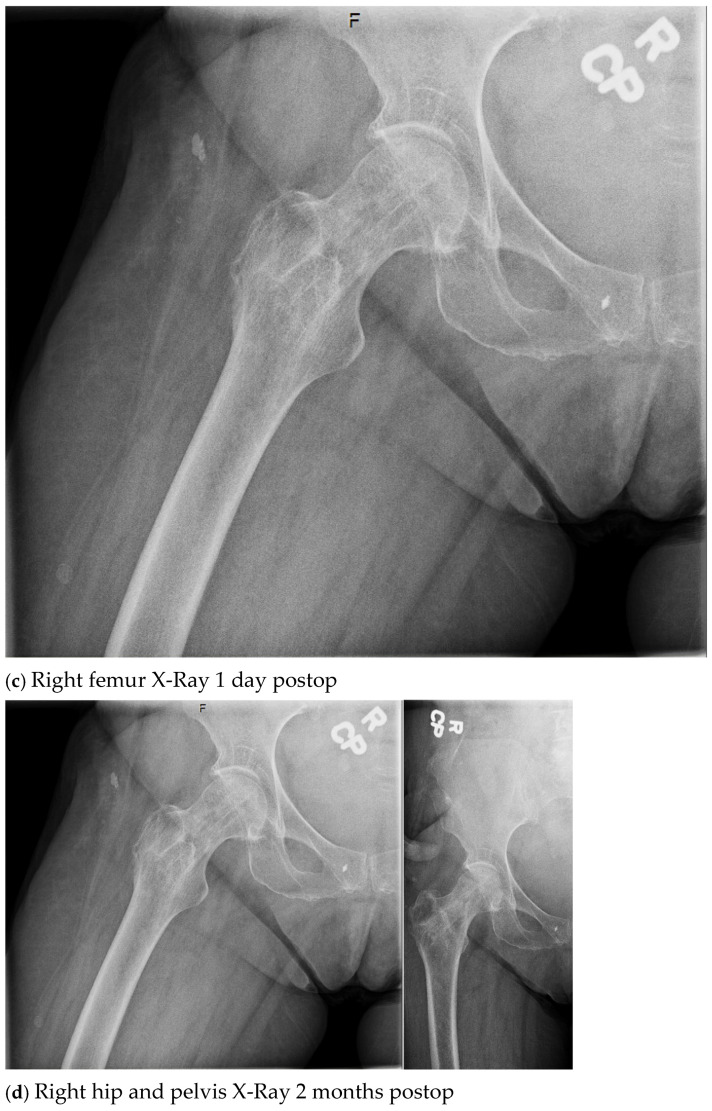

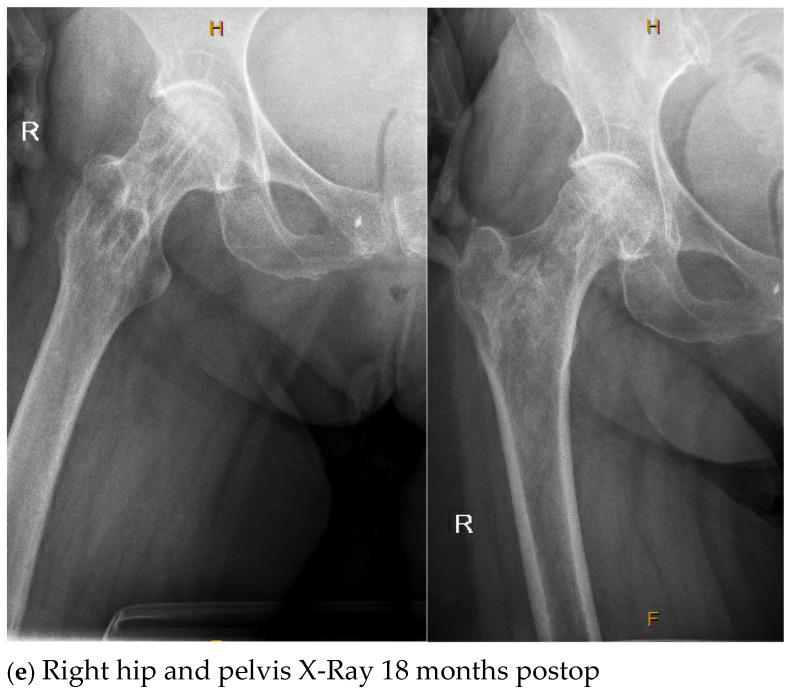
X-Ray images depicting surgical management (Case 4).

All surgical procedures were performed by the senior author and performed in standard fashion, which included cultures of the infected hardware with multiple specimens, usually five, being sent to microbiology with sampling of the soft tissue and the bone involved. A high-speed burr was used to resect infected bone, and, in case of an intermedullary canal infection, sequential reaming or a reamer irrigator aspirator was used. Once the bone was clean, bleeding, and healthy, the wound and bone were irrigated with 6 L of saline, and the affected limb was prepped and redraped. CaS/HA-G with or without vancomycin/Tobramycin was then mixed on the back table in standard fashion, with standard doses of Gentamicin, Vancomycin, or Tobramycin; this dosing was 1.0 g, 0.5 g, and 1.2 g per 10 cc of CaS/HA-G. This Cerement was then injected into a dried surgical bone bed, and a lap sponge was held over the top of this until it got hard. Following this, the wound was closed in standard fashion with or without a wound VAC application on the incision. Occasionally, a Gastrocnemius muscle flap was used in the case of the anterior and medial tibia, where the soft tissue coverage was poor. A sample of the X-Ray images depicting surgical management is shown in Figure 2.

The rationale for using the absorbable antibiotic depot CaS/HA-G was based on the presence of a contained bone void defect. The amount of CaS/HA-G applied was sufficient to completely fill the defect. In cases where Gram-positive organisms were identified preoperatively, vancomycin was added as an adjunct therapy.

Patients were required to have a follow-up every two weeks and any wound drainage was monitored carefully. CRP and ESR were requested weekly, and patients were placed on postoperative IV antibiotics for six weeks, which were appropriate for the cultured organism.

### 5.4. Infection Eradication

The diagnosis of infection eradication was determined by either the normalization of ESR and CRP levels or the absence of clinical infection symptoms. Normalization was defined as ESR and CRP levels falling under the maximum threshold of the reference range specific to each patient based on their baseline levels. Clinical indicators of the absence of infection included lack of pain, swelling, redness, and discharge from the surgical site.

### 5.5. Statistical Analysis

Descriptive statistics were calculated for numerical variables, including the mean and standard deviation or median and interquartile range. Frequencies and percentages were computed for categorical variables, such as the eradication rate. All analyses were performed using Statistical Product and Service Solution (SPSS, version 28.0, IBM Corp., Armonk, NY, USA).

## Figures and Tables

**Table 1:** Baseline characteristics of patients and details of antibiotics used.

Case	Age (Years)	Sex	Diabetes Mellitus	Smoking	CaS/HA-G Quantity (cc)	Vancomycin (g)	Tobramycin (g)	Follow-Up (Months)	Diagnosis
1.	40	F	N	N	60	N	N	19	Infected right distal tibia with bone defect and open wound
2.	51	M	N	N	10	0.5	N	17	Left humeral osteomyelitis with retained plate
3.	20	F	N	N	40	2.0	N	17	Left femoral osteomyelitis
4.	75	F	Y	N	20	N	N	17	Right thigh and gluteal abscess with infected right femoral neck hardware and osteomyelitis
5.	70	F	N	N	20	1.0	2.4	16	Failed right total knee arthroplasty with infection
6.	52	F	N	N	20	1.0	2.4	16	Right femoral osteomyelitis
7.	18	M	N	N	30	1.5	N	15	Left anterior tibial osteomyelitis with soft tissue defect
8.	34	M	N	N	20	N	N	14	Nonunion left femur
9.	37	M	N	Y	60	3.0	N	8	Right femur osteomyelitis
10.	63	F	N	N	30	1.5	N	7	Right tibial infected hardware and osteomyelitis
11.	57	M	N	N	50	2.5	N	7	Right distal osteomyelitis with draining sinus
12.	54	M	N	N	30	1.5	N	6	Infected right knee arthrodesis rod, peroneal nerve entrapment right leg
13.	67	M	Y	N	20	1.0	2.4	10	Infected right revision long stemmed total knee arthroplasty with deficient anterior soft tissue envelope, right knee fusion nonunion
14.	71	M	N	N	10	0.5	1.2	10	Right infected ankle open reduction internal fixation with osteomyelitis
15.	53	F	Y	N	10	0.5	N	10	Painful prominent right foot hardware with infected midfoot nonunion
16.	67	M	N	N	65	3.5	N	13	Left tibial osteomyelitis with infected spacer and medial tibial open wound
17.	66	M	N	N	10	N	N	10	Infected right elbow hardware with olecranon osteomyelitis and chronic draining sinus
18.	63	F	N	N	20	1.0	2.4	13	Right tibia periprosthetic open fracture
19.	67	F	N	Y	10	0.5	N	6	Left infected ankle fusion nonunion with retained hardware
20.	73	M	Y	N	15	0.75	N	6	Right knee proximal tibial retained hardware
21.	52	M	N	N	40	2.0	4.8	7	Right tibial chronic osteomyelitis

F, Female; M, Male; N, No; Y, Yes; TKA, Total knee arthroplasty; ORIF, Open reduction and internal fixation; NA, Not available.

**Table 2:** Treatment details and outcomes of osteomyelitis patients treated with CaS/HA.

Case	Reinfection	Reoperation (Weeks Post 1st Surgery)	ESR (mm/h)	CRP (mg/L)	Infection Eradication	Eradication Time (Days)	Culture
1.	Y	Y (6)	2	14	Y, clinical and lab	76	1st Op: *Staphylococcus aureus* 2nd Op: *Serratia marcescens*, *Enterococcus faecalis*
2.	N	N	NA	NA	Y, clinical	61	*Pseudomonas* species
3.	N	N	29	3	Y, clinical	232	*Staphylococcus aureus*
4.	N	N	14	4	Y, clinical and lab	107	Negative
5.	-	-	43	270	N	-	Negative
6.	N	N	27	3	Y, clinical and lab	63	Negative
7.	N	Y (3)	3	<1	Y, clinical and lab	272	1st Op: *Staphylococcus aureus*, *Klebsiella* (*Enterobacter*) *aerogenes* 2nd Op: Negative
8.	N	N	NA	NA	Y, clinical	428	Negative
9.	N	N	13	<1	Y, clinical and lab	160	Negative
10.	N	N	26	1	Y, clinical and lab	144	Methicillin Resistant *Staphylococcus aureus*
11.	Y	Y (17)	28	1	Y, clinical and lab	202	Negative
12.	N	N	33	6.5	Y, clinical	99	Methicillin Resistant *Staphylococcus aureus*
13.	Y	Y (17)	15	4	Y, clinical and lab	234	1st Op: *Corynebacterium striatum* 2nd Op: *Serratia marcescens*
14.	N	N	16	5	Y, clinical and lab	319	Methicillin Resistant *Staphylococcus aureus*
15.	N	N	3	2	Y, clinical and lab	117	*Streptococcus viridans*, *Finegoldia magna*, *Staphylococcus* coag negative, *Streptococcus mitis/Streptococcus oralis*, *Corynebacterium jeikeium*, *Corynebacterium* species
16.	N	N	2	<1	Y, clinical and lab	139	Methicillin Resistant *Staphylococcus aureus*
17.	N	N	24	1	Y, clinical and lab	51	*Staphylococcus aureus*
18.	N	N	19	<1	Y, clinical and lab	385	*Staphylococcus* coag negative
19.	N	N	126	34	Y, clinical	74	*Staphylococcus aureus*
20.	N	N	NA	NA	Y, clinical and lab	27	*Proteus mirabilis*, *Enterococcus faecalis*, *Prevotella bivia*, Beta Lactamase Positive
21.	N	N	23	2	Y, clinical and lab	71	*Staphylococcus aureus*

ESR, erythrocyte sedimentation rate; CRP, C-reactive protein; Y, Yes; N, No; NA, Not available.

## Data Availability

Data access is available upon reasonable request by contacting the corresponding author.
